# A [^14^C]iodoantipyrine study of inter-regional correlations of neural substrates following central post-stroke pain in rats

**DOI:** 10.1186/s12990-015-0006-5

**Published:** 2015-03-08

**Authors:** Hsiang-Chin Lu, Wei-Jen Chang, Yung-Hui Kuan, Andrew Chih-Wei Huang, Bai Chuang Shyu

**Affiliations:** Institute of Biomedical Sciences, Academia Sinica, Taipei, 11529 Taiwan; Department of Psychology, Fo Guang University, Yilan, 26247 Taiwan

**Keywords:** Central post-stroke pain, Autoradiography, Isotope, Brain circuits, Spinothalamic tract, Medial thalamus, Anterior cingulate cortex, mPFC-amygdala pathway

## Abstract

**Background:**

Central pain syndrome is characterized by a combination of abnormal pain sensations, and pain medications often provide little or no relief. Accumulating animal and clinical studies have shown that impairments of the spinothalamic tract (STT) and thalamocingulate pathway causes somatosensory dysfunction in central post-stroke pain (CPSP), but the involvement of other neuronal circuitries in CPSP has not yet been systematically examined. The aim of the present study was to evaluate changes in brain activity and neuronal circuitry using [^14^C]iodoantipyrine (IAP) in an animal model of CPSP.

**Results:**

Rats were subjected to lateral thalamic hemorrhage to investigate the characteristics of CPSP. Thermal and mechanical hyperalgesia developed in rats that were subjected to thalamic hemorrhagic lesion. The medial prefrontal cortex (mPFC), anterior cingulate cortex (ACC), striatum, thalamus, hypothalamus, and amygdala were more active in the CPSP group compared with rats that were not subjected to lateral thalamic hemorrhage. The inter-regional correlation analysis showed that regional cerebral blood flow in the mPFC was highly correlated with the amygdala in the right brain, and the right brain showed complex connections among subregions of the ACC. Rats with CPSP exhibited strong activation of the thalamocingulate and mPFC-amygdala pathways.

**Conclusions:**

These results corroborate previous findings that the STT and thalamocingulate pathway are involved in the pathophysiological mechanisms of CPSP symptoms. The mPFC, amygdala, and periaqueductal gray emerged as having important correlations in pain processing in CPSP. The present data provide a basis for a neural correlation hypothesis of CPSP, with implications for CPSP treatment.

## Background

Many clinical symptoms are manifested after stroke, including motor deficits, cognitive dysfunction, language problems, emotional disturbances, social maladjustment, somatosensory dysfunction, and central pain [1,2]. More than 8% of stroke patients have neuropathic pain, termed central post-stroke pain (CPSP) [3]. CPSP might result from many somatosensory dysfunctions, including hypersensitivity and allodynia. Some hypotheses of the pathophysiological mechanisms of somatosensory dysfunction have been proposed to explain CPSP symptoms, such as the loss of somatic sensations caused by the deafferentation of neurons in the hemorrhagic area, hyperalgesia that results from the hyperexcitability of central nociceptive neurons, and central disinhibition [4,5]. These pathophysiological mechanisms of CPSP provide some insights for determining the possible neural substrates of CPSP.

Several neural substrates, including cerebral activity, might be involved in the pathophysiology of CPSP [6-8]. For example, a recent study showed that the expression of interleukin-1β in the hippocampus, prefrontal cortex, and brainstem may be correlated with chronic neuropathic pain-like behavior [9]. A review article reported that the descending pain modulation system, including the dorsolateral prefrontal cortex, rostral anterior cingulate cortex (ACC), amygdala, hippocampus, periaqueductal gray (PAG), and rostral ventromedial medulla, comprises a network that regulates nociceptive processing [10]. The lateral and medial pain pathways have been showed to govern the homeostasis of nociception processing in coding the intensity of pain [8,11-13]. Stroke patients with dysfunction in the lateral thalamus exhibited a disruption of inhibition of signaling to the medial thalamus (MT), resulting in mechanical allodynia and thermal hyperalgesia [6,7]. CPSP of thalamic origin can be viewed as a disinhibition disorder associated with thermoregulatory integration. Additionally, the ventral posteromedial thalamic nucleus (VPM)-dorsal posterior insular pathway has been shown to inhibit pain processing in limbic networks that consist of the MT, ACC, and PAG, suggesting that CPSP may be attributable to the loss of the aforementioned inhibition [14,15].

Human functional magnetic resonance imaging (fMRI) studies have provided important evidence that the spinothalamic tract (STT) and MT-ACC pathway might be involved in CPSP [15-18]. For example, brain lesions of the lateral and posterior thalamus and ventral nucleus-pulvinar border zone have been shown to be associated with a higher risk of developing CPSP after thalamic insult [15]. A recent correlational study used clinical quantitative sensory testing (QST), MRI, and single-photon emission computed tomography (SPECT) and found that MRI and SPECT images of thalamic and parietal cortex lesions were correlated with CPSP-related allodynia symptoms in QST tests [16]. A recent human fMRI study dissociated differences in thalamic subregions (e.g., ventral posterolateral [VPL] and posterior portion [VMpo] of the ventral medial nucleus) in CPSP, indicating that the VPL but not VMpo plays a crucial role in CPSP [17]. Furthermore, another fMRI study indicated that the contralateral somatosensory cortex and bilateral mid/posterior insula, anterior insula, and posterior cingulate were activated during exposure to acute pain stimulation [18]. Therefore, the STT and MT-ACC pathway may be critically involved in CPSP symptoms.

A growing body of evidence indicates that the activation of medial prefrontal cortex (mPFC)-to-amygdala circuitry is involved in pain-related emotion and governs nociception in rodents [19-23]. Therefore, the present study evaluated whether the mPFC-amygdala pathway, STT, MT-ACC pathway, and other neural substrates are involved in CPSP.

Brain responses in CPSP patients have been investigated using functional brain mapping [15,16,24]. Functional magnetic resonance imaging measures global neuronal activity in response to specific stimuli. Positron emission tomography (PET) is another brain mapping approach that determines active brain localization using a radioactive substance. Functional magnetic resonance imaging and PET are powerful tools with high spatial resolution for evaluating brain activity. Moreover, fMRI can provide real-time brain mapping data. However, the costs for fMRI and PET are high and/or require anesthesia. In contrast, the [^14^C]iodoantipyrine (IAP) uptake method requires no anesthesia and is less expensive. The [^14^C]IAP uptake method also has some limitations. It involves the indirect observation of regional cerebral blood flow (rCBF) by assuming a brain activity-cerebral blood flow relationship. The most critical symptom associated with CPSP is the sensation of constant burning (i.e., spontaneous pain) [25]. Thus, to spontaneously measure the brain events that occur in a pathological state, the [^14^C]IAP uptake method is suitable for mapping brain activity during spontaneous pain.

In human studies, hypersensitivity and allodynia associated with CPSP have been shown to be important symptoms [1]. A novel animal model of CPSP has been developed recently, suggesting that lesions of the ventral basal nucleus (VB) of the thalamus can cause hypersensitivity and allodynia in CPSP [26]. No animal studies have systematically and comprehensively investigated alterations in brain activity related to hypersensitivity and allodynia in an animal model of CPSP. The aim of the present study was to utilize [^14^C]IAP radioactivity to evaluate brain activity and circuitry changes after lesions of the VB in rats. Furthermore, some neural circuits, including the STT and MT-ACC pathway, mPFC-amygdala pathway, and other neural substrates, were examined to investigate the neural circuitries that are involved in brain activation associated with CPSP.

## Results

### Correlations between [^14^C]IAP radioactivity and imaging signals

**Figure 1:**
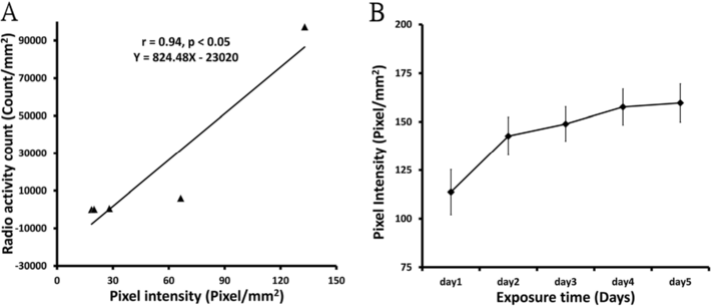
**Standard curves of autoradiography. A**. Correlations between pixel intensity and pixel count. The pixel intensity was highly correlated with pixel count. **B**. Pixel intensity increased with increasing exposure time. The optimal resolution appeared on day 4.

Brain activity was correlated with [^14^C]IAP signal intensity in brain images. The pixel intensity of brain images using the [^14^C]IAP method can elucidate metabolism in the brain and allow quantitative analyses. To quantify the pixel intensity of images of different brain slices, image normalization is very important. We first defined the range of signal intensities to be included in the image analysis. The range of the environmental background signal was 25.811-46.979 counts per minute (CPM), and the signal of the [^14^C]IAP 0.001 μCi filter paper was 42 CPM. Therefore, pixels with a signal intensity < 0.001 μCi were considered background. Five dose ranges (0.001-10 μCi ^14^C) could be displayed as gray-scale pixels on the images. The doses of [^14^C]IAP radioactivity were positively correlated with the pixel intensity and radioactivity count on a logarithmic scale. The pixel intensity was also shown to be positively correlated with radioactivity count (*r* = 0.94, *p* < 0.05), with a linear regression equation was obtained between the pixel intensity and radioactivity count, (Equation 1, Figure 1A).

1 $$ \left(Y = 824.48\ \mathrm{X} - 23020\right) $$

The pixel intensity was positively correlated with exposure time. The intensity increased from day 1 to day 5. However, the pixel intensity was not significantly different (*p* > 0.05) between day 4 and day 5. Thus, 4 days was determined to be the optimal exposure time for [^14^C]IAP to obtain maximal pixel intensity (Figure 1B).

### Behavioral hyperalgesia occurred after thalamic lesion

**Figure 2:**
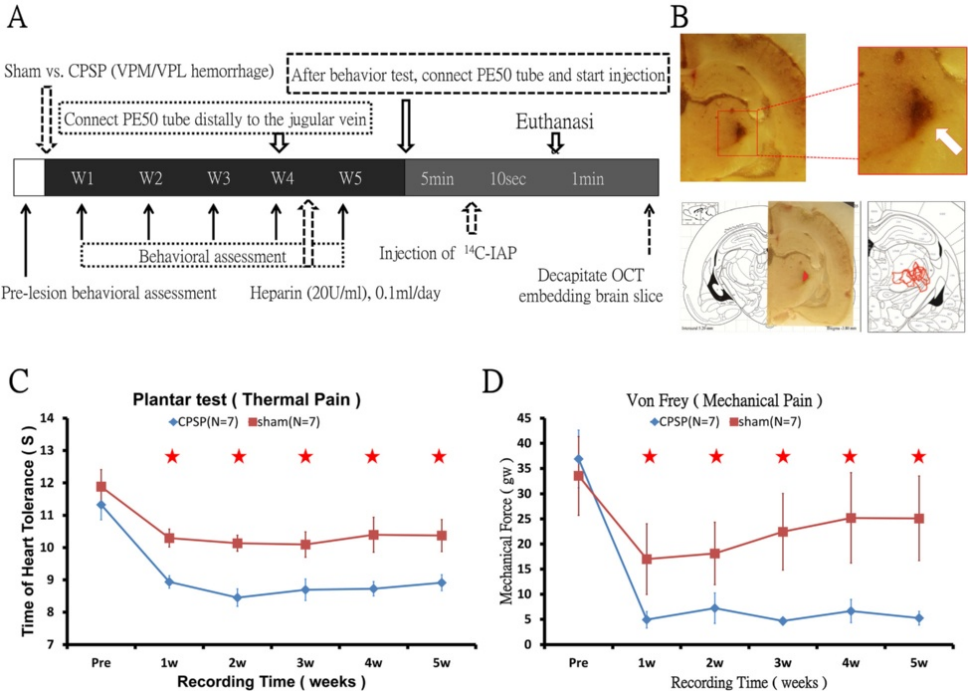
**Experimental timeline and example of mechanical and thermal hyperalgesia after lesion of the VPL and VPM. A**. Experimental timeline of [^14^C] experiment. **B**. Example of collagenase-induced lesion in the VPL and VPM 1 month after injection. **C** and **D**. Plantar test and von Frey test on the left hindpaw in the sham and CPSP groups on day 35. *p* < 0.05 (two-way ANOVA followed by *post hoc* test). The results showed that the CPSP group had lower pain thresholds than the sham group.

The experimental timeline for the brain lesions, behavioral measurements, and brain imaging is illustrated in Figure 2A. A typical collagenase-induced lesion in the VPL and VPM in frozen brain slices is shown in Figure 2B (white arrow). The lesion sites from all of the histological samples are encircled with red lines and overlap in the corresponding brain atlas. The lesion sites were all confirmed and localized in the VB area (Figure 2B).

To test the behavioral influence of thalamic lesions on thermal nociception and mechanical pain threshold, sham and CPSP rats were evaluated using the plantar test and von Frey test. The plantar test was performed to determine changes in thermal nociception. A 2 × 5 mixed two-way analysis of variance (ANOVA) indicated that thermal pain threshold in the CPSP group was significantly decreased compared the sham group (*F*_1,40_ = 45.86, *p* < 0.05). The significant difference between groups lasted from week 1 to week 5 of the experiment (Tukey’s Honestly Significant Difference (HSD) *post hoc* test, all *p* < 0.05; Figure 2C). No significant difference in pain threshold was found between the different weeks after collagenase or saline treatment in each group (*F*_4,160_ = 0.43, *p* > 0.05).

Changes in mechanical pain thresholds were assessed in the von Frey test. Mechanical thresholds in the CPSP group were significantly lower than in the sham group (2 × 5 mixed two-way ANOVA; *F*_1,40_ = 15.55, *p* < 0.05). A significant group × lesion weeks interaction was observed (*F*_4,160_ = 2.81, *p* < 0.05). CPSP rats exhibited significant changes in mechanical hyperalgesia from week 1 to week 5 (Tukey’s HSD *post hoc* test, all *p* < 0.05; Figure 2D). No significant difference in pain thresholds was found between the different weeks after collagenase or saline treatment in each group (*F*_4,160_ = 2.67, *p* > 0.05).

### Brain imaging of [^14^C]IAP

**Figure 3:**
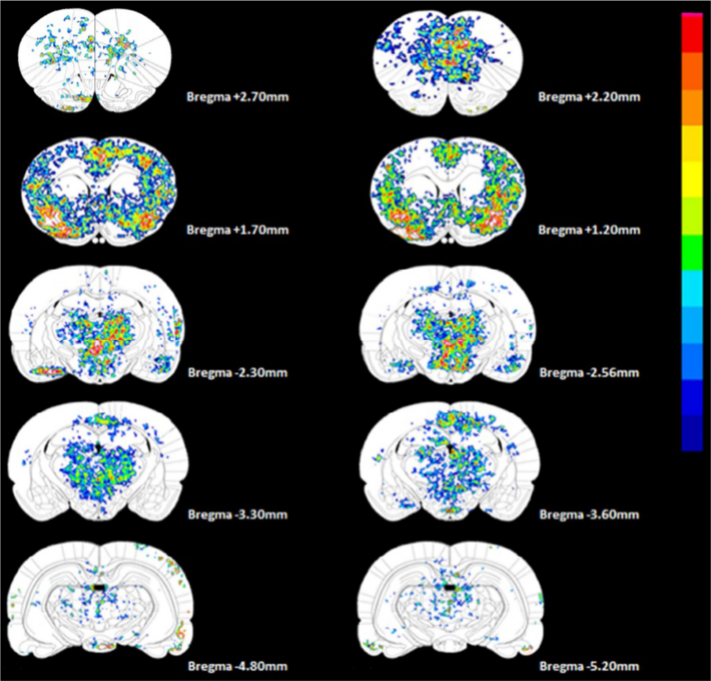
**Key brain regions that showed significant differences in pixel counts between the CPSP and sham groups.** The results were obtained by differential subtraction between the CPSP and sham groups. The signals in the cortex, thalamus, hypothalamus, and amygdala in the CPSP group were significantly higher than in the sham group, and the signal in the ventral basal nucleus in the CPSP group was significantly lower than in the sham group.

To examine the brain areas that are involved in CPSP symptoms and their signal intensity, Statistical Parametric Mapping (SPM) was used. Whole-brain images were composed of a sequence of images in a coronal section that were normalized and analyzed by SPM. The differences between the sham and CPSP groups were indexed by subtracting the sham group from the CPSP group. Pixel counts were significantly different (*p* < 0.05) and are presented in pseudo color in Figure 3. Compared with the sham group, the CPSP group showed significant alterations in the pixel count ratio, reflecting [^14^C]IAP expression in some major brain regions, including the cortex, striatum, thalamus, hypothalamus, and amygdala.

To quantitatively analyze the SPM results, a 2 × 31 × 2 three-way (group *vs*. brain area *vs*. hemisphere) ANOVA was performed, indicating significant effects of group (*F*_1,744_ = 2492.36, *p* < 0.05) and brain area (*F*_30,744_ = 173.66, *p* < 0.05) and a significant group × brain area interaction (*F*_30,744_ = 98.34, *p* < 0.05). No significant effect of hemisphere was found (*F*_1,744_ = 0.00, *p* > 0.05), with no group × hemisphere interaction (*F*_1,744_ = 0.08, *p* > 0.05), brain area × hemisphere interaction (*F*_30,744_ = 0.45, *p* > 0.05), or group × brain area × hemisphere interaction (*F*_30,744_ = 0.42, *p* > 0.05). Furthermore, a 2 × 31 two-way (group *vs*. brain area) ANOVA indicated significant effects of group (*F*_1,806_ = 2608.01, *p* < 0.05) and brain area (*F*_30,806_ = 181.72, *p* < 0.05) and a significant group × brain area interaction (*F*_30,806_ = 102.90, *p* < 0.05). The *post hoc* Tukey’s HSD test indicated that the pixel counts in several brain regions were significantly different between the CPSP and sham groups (all *p* < 0.05), such as the cingulate cortex area 1 (Cg1), somatosensory cortex (S1), granular insular cortex (GI), hippocampus (Hippo), thalamus (e.g., mediodorsal thalamic nucleus [MD], VB, ventrolateral thalamic nucleus [VL], and ventromedial thalamic nucleus [VM]), hypothalamus (e.g., lateral hypothalamic area [LH] and ventromedial hypothalamic nucleus [VMH]), amygdala, and PAG. Overall, [^14^C] IAP was high uptake in the mPFC and ACC in the CPSP group and showed the strongest [^14^C] signals in the brain images. Significant differences in radioactivity in many brain areas are listed in Table 1.

**Table 1:** **Key brain regions that show significant changes in [** ^**14**^ **C] signals using two different brain imaging methods**

**Brain region**	**SPM**	**ROI**	
	**Left/Right**	**Left/Right**	
Infralimbic cortex (IL)	P = 1.00	P = 0.02*	P = 0.00*
Prelimbic cortex (PrL)	P = 1.00	P = 0.30	P = 0.00*
Cingulate cortex area 2 (Cg2)	P = 0.28	P = 0.60	P = 1.00
Cingulate cortex area 1 (Cg1-1)	P = 1.00	P = 0.41	P = 0.00*
Cingulate cortex area 1 (Cg1-2)	P = 0.02*	P = 0.04*	P = 0.36
Secondary motor cortex (M2-1)	P = 1.00	P = 1.00	P = 0.87
Secondary motor cortex (M2-2)	P = 0.28	P = 0.04*	P = 0.00*
Primary motor cortex (M1-1)	P = 1.00	P = 1.00	P = 0.86
Primary motor cortex (M1-2)	P = 0.58	P = 0.38	P = 0.88
Somatosensory cortex (S1-1)	P = 1.00	P = 0.93	P = 0.65
Somatosensory cortex (S1-2)	P = 0.53	P = 0.22	P = 0.98
Somatosensory cortex (S1-3)	P = 0.00*	P = 1.00	P = 1.00
Somatosensory cortex (S1-4)	P = 0.00*	P = 1.00	P = 1.00
Granular insular cortex (GI-1)	P = 1.00	P = 0.08	P = 0.34
Granular insular cortex (GI-2)	P = 0.82	P = 0.88	P = 0.85
Granular insular cortex (GI-3)	P = 0.00*	P = 1.00	P = 0.90
Agranular insular dorsal cortex (AID)	P = 1.00	P = 0.00*	P = 0.39
Agranular insular ventral cortex (AIV)	P = 1.00	P = 0.03*	P = 0.52
Striatum	P = 0.07	P = 1.00	P = 1.00
Hippocampus (Hippo-1)	P = 0.00*	P = 1.00	P = 1.00
Hippocampus (Hippo-2)	P = 0.00*	P = 1.00	P = 1.00
Hippocampus (Hippo-3)	P = 0.01*	P = 0.00*	P = 0.14
Mediodorsal thalamus nucleus (MD)	P = 0.00*	P = 0.54	P = 0.99
Ventral basal nucleus (VB)	P = 0.00*	P = 0.97	P = 0.00*
Ventrolateral thalamus nucleus (VL)	P = 0.00*	P = 1.00	P = 1.00
Ventromedial thalamus nucleus (VM)	P = 0.00*	P = 1.00	P = 1.00
Lateral hypothalamic area (LH)	P = 0.00*	P = 1.00	P = 1.00
Ventral medial hypothalamus nucleus (VMH)	P = 0.00*	P = 1.00	P = 1.00
Amygdala (Amygdala-1)	P = 0.00*	P = 1.00	P = 1.00
Amygdala (Amygdala-2)	P = 0.00*	P = 0.10	P = 0.01*
Periaqueductal gray (PAG)	P = 0.00*	P = 0.00*	P = 0.50

Significant increases or decreases in [^14^C] uptake are noted with p values, respectively, for the SPM and ROI image signals. *p < 0.05.

### Region-of-interest analysis

**Figure 4:**
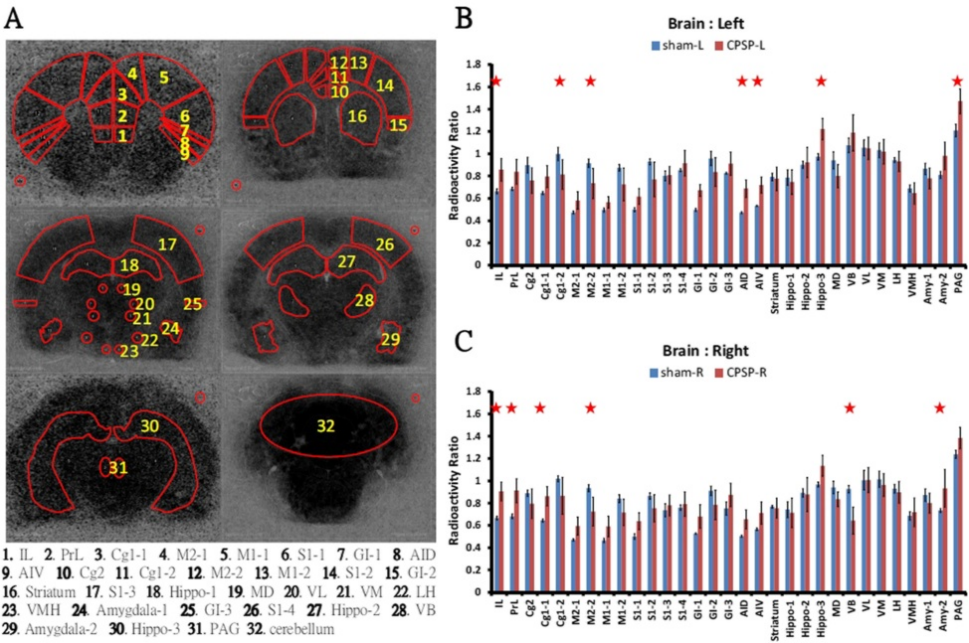
**Region-of-interest (ROI) analysis. A**. The boundaries of the ROIs were chosen based on available anatomic atlases. **B**. In the comparison of the relative ratios of the left hemisphere ROIs in the different groups, the radioactivity ratios of the IL, GI-3, AID, AIV, Hippo-3, and PAG in the CPSP group were significantly higher than in the sham group, and the Cg1-2 and M2-2 in the CPSP group were significantly lower than in the sham group. **C.** In the comparison of the relative ratios of the right hemisphere ROIs in the different groups, the radioactivity ratios of the IL, PrL, Cg1-1, and Amy-2 in the CPSP group were significantly higher than in the sham group, and the M2-2 and VB were significantly lower. *p* < 0.05 (three-way ANOVA followed by *post hoc* test).

To further evaluate changes in brain areas associated with CPSP symptoms, a region-of-interest (ROI) analysis was conducted. Different brain areas related to CPSP were distinguished and quantitatively analyzed. The ROI analysis was conducted based on the results of the functional brain images of [^14^C]-IAP uptake shown in Figure 3. The assignment of the ROIs for specific brain areas is shown in Figure 4A. A 2 × 31 × 2 three-way (group *vs*. brain area *vs*. hemisphere) ANOVA was used for the analysis, which revealed significant main effects of group (*F*_1,744_ = 29.55, *p* < 0.05), brain area (*F*_30,744_ = 119.12, *p* < 0.05), and hemisphere (*F*_1,744_ = 18.05, *p* < 0.05), a group × brain area interaction (*F*_30,744_ = 17.44, *p* < 0.05), a brain area × hemisphere interaction (*F*_30,744_ = 4.94, *p* < 0.05), and a group × brain area × hemisphere interaction (*F*_30,744_ = 1.88, *p* < 0.05). No group × hemisphere interaction was found (*F*_1,744_ = 0.30, *p* > 0.05).

A 2 × 31 two-way ANOVA (group *vs*. brain area) was performed to analyze ROI scores, which revealed significant main effects of group (*F*_1,806_ = 24.64, *p* < 0.05) and brain area (*F*_30,806_ = 99.31, *p* < 0.05) and a group × brain area interaction (*F*_30,806_ = 14.54, *p* < 0.05). The specific ROIs in the sham control and CPSP groups in the left and right hemispheres were measured and analyzed using Tukey’s HSD *post hoc* test. In the left hemisphere, significant differences were found in the infralimbic cortex (IL), Cg1, M2, dorsal agranular insular cortex (AID), ventral agranular insular cortex (AIV), Hippo-3, and PAG between the sham and CPSP groups (all *p* < 0.05; Figure 4B). In contrast, the right hemisphere showed significant differences in the IL, prelimbic cortex (PrL), Cg1-1, VB, and amygdala-2 (all *p* < 0.05; Figure 4C). Major differences in [^14^C]-IAP signals were found between the CPSP and sham groups in the cortex, thalamus, hypothalamus, amygdala, and PAG. These results suggest that the cortex on both sides of the brain might be involved in CPSP, and the hippocampus and PAG in the left hemisphere were activated solely in the CPSP group. The VB of the right thalamus exhibited a reduction, and this reduction appeared to result from the hemorrhagic lesion. The significant differences in the ROI analysis are presented in Table 1.

### ROI vs. behavior correlation analysis

To evaluate whether these brain regions showed significant differences that were linked with nociceptive behavioral responses after the thalamic hemorrhagic lesion, Pearson correlations were conducted for pain-related behavioral responses and selected brain mapping ROIs that exhibited significant differences with the SPM and ROI scores in the ANOVA. The sham group did not exhibit correlations between the ROIs and plantar test results (*r* = 0.01, *p* > 0.05) or von Frey test results (*r* = 0.01, *p* > 0.05). In contrast, in the CPSP group, significant negative correlations were observed between the ROIs and plantar test results (*r* = −0.13, *p* < 0.05), with no correlation between the ROIs and von Frey test results (*r* = 0.09, *p* > 0.05; Table 2). The results of these two behavioral tests showed significant positive correlations in the sham group (*r* = 0.50, *p* < 0.05) and significant negative correlations in the CPSP group (*r* = −0.26, *p* < 0.05), indicating that these two behavioral tests presented the same trend for pain. A negative correlation was found between pain behavior and the imaging data, suggesting that changes in brain activity after CPSP are more related to changes in thermal nociceptive sensitivity.

**Table 2:** **Pearson correlations between behavioral tests and ROI assessment over selected brain areas**

**Group**	**Correlation test**	**Result**
*Sham group*	Plantar test *vs*. ROIs	*r* = 0.01, *p* > 0.05
	von Frey *vs*. ROIs	*r* = 0.01, *p* > 0.05
	Plantar test vs. von Frey	*r* = 0.50, *p* < 0.05*
*CPSP group*	Plantar test *vs*. ROIs	*r* = −0.13, *p* < 0.05*
	von Frey *vs*. ROIs	*r* = 0.09, *p* > 0.05
	Plantar test vs. von Frey	r = −0.26, *p* < 0.05*

*p < 0.05, significant difference.

### Pair-wise interregional correlation analysis

**Figure 5:**
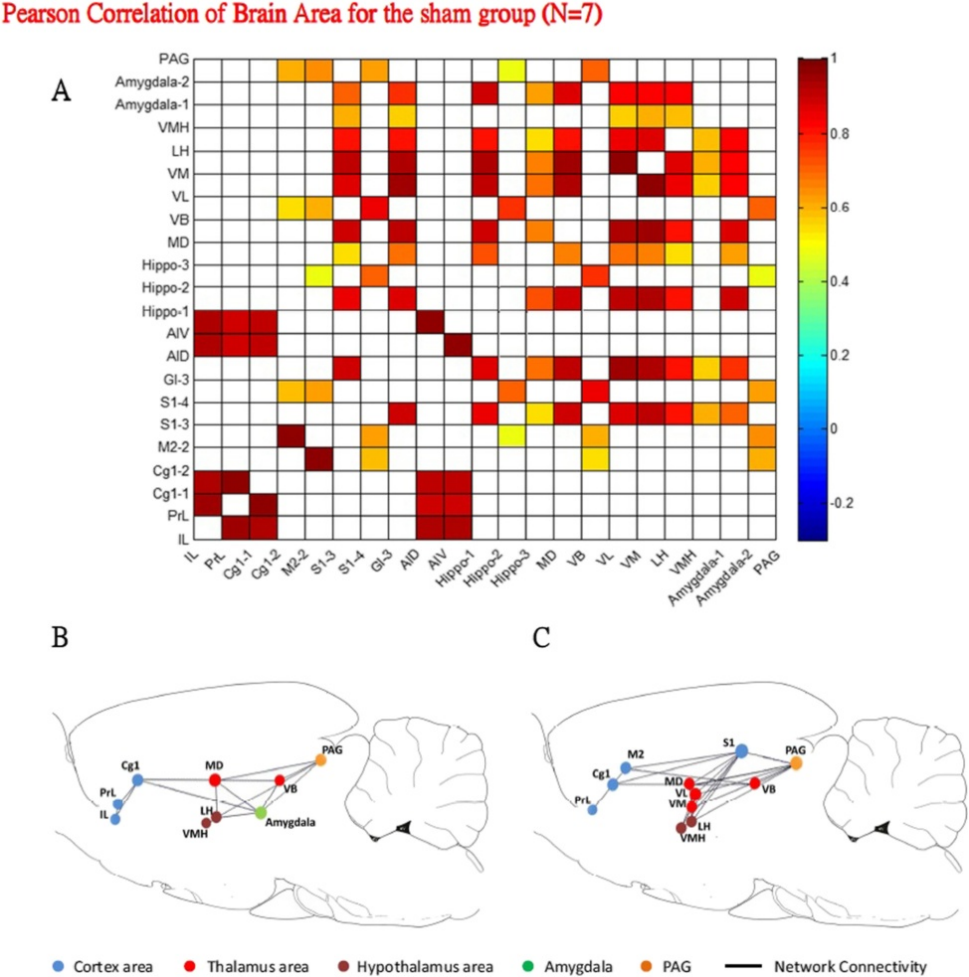
**Inter-regional rCBF in sham rats was analyzed by assessing correlations.** Twenty-two ROIs that showed significant increases in radioactivity counts in sham rats were included into the correlation analysis. **A**. Inter-regional correlation matrix for the sham group. The matrix is symmetrical across the diagonal line from the lower left to the upper right. Notice the strong positive connections among cortical ROIs, including the cortex (IL, PrL, Cg1-1, Cg1-2, S1-3, AID, and AIV) and thalamus (MD, VL, and VM). The connections among the VB, hypothalamus (LH and VMH), Amygdala (Amygdala-1 and Amygdala-2), and PAG were weakly positive with this corticostriatal cluster. **B**. Inter-regional correlations of rCBF of the mPFC-amygdala pathway in the sham group. The vertices (colored dots) represent ROIs of different brain areas. The connectivity map showed connections between vertices with significant correlations. **C**. Graphical representation of the inter-regional correlations of rCBF of the MT-ACC pathway and STT in the sham group.

**Figure 6:**
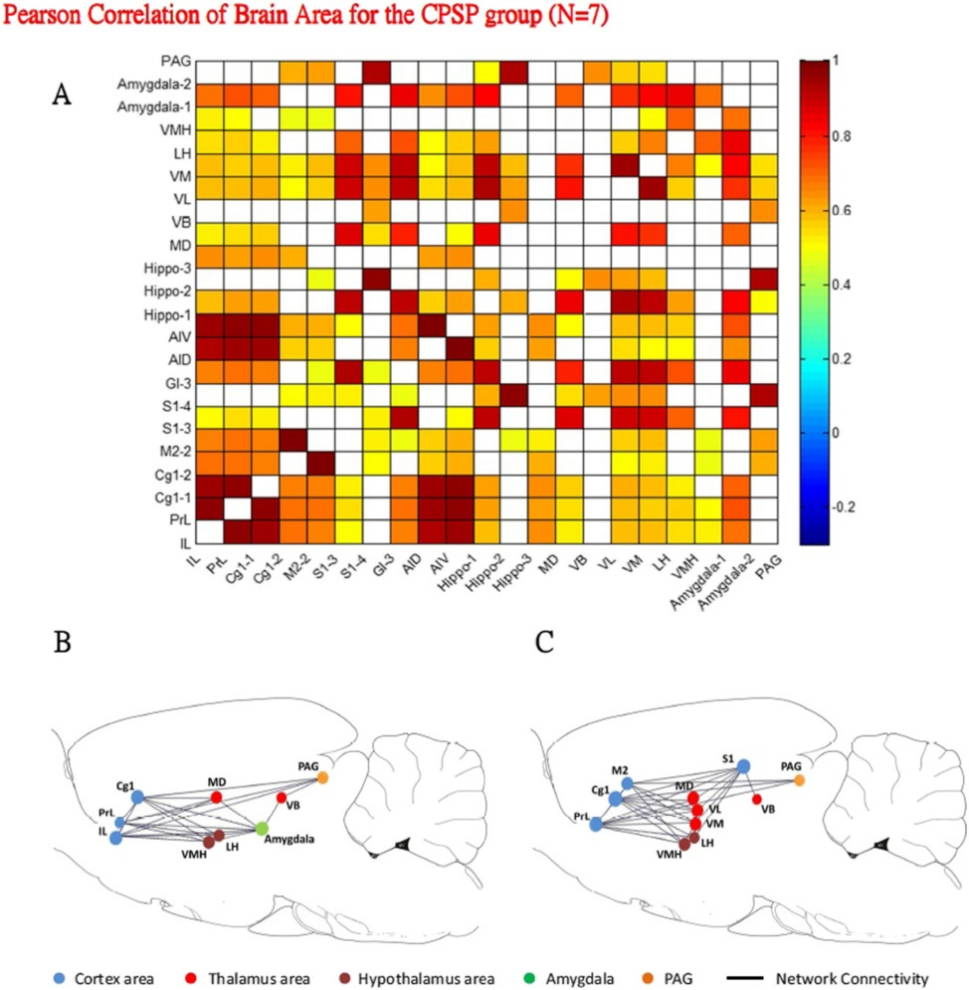
**Inter-regional rCBF in CPSP rats was analyzed by assessing correlations.** Twenty-two ROIs that showed significant increases in radioactivity counts in the CPSP group were included in the correlation analysis. **A**. Inter-regional correlation matrix of inter-regional rCBF patterns in the CPSP group. Notice the augmented number of significant connections. The thalamus and hypothalamus were much more strongly correlated with the cortex compared with the sham group (see Figure 5A). **B**. The inter-regional correlation of the mPFC-amygdala pathway in the CPSP group is represented by a graph in which the connections between vertices with significant correlation are shown. The areas of the mPFC were much more positively correlated with the MD and amygdala compared with the sham group (see Figure 5B) **C**. Graphical representation of inter-regional correlations of the MT-ACC pathway and STT in the CPSP group. The cortex, thalamus, and hypothalamus had more connections with each other.

The ROI data of the specific brain areas that exhibited significant differences with the SPM and ROI scores in the ANOVA were further analyzed using Pearson correlations. The interregional correlation matrices of the ROIs in the sham and CPSP groups are depicted in Figures 5A and 6A, respectively. Major brain areas, such as the cortex, hippocampus, thalamus, hypothalamus, amygdala, and PAG, were selected for analysis. Significant functional correlations were found between different brain regions in the interregional correlation matrix (*p* < 0.01). The diagonal line from the lower left to upper right of the matrix is symmetrical, indicating correlations between ROIs and themselves. The analysis identified 154 (33.3%) significant positive correlations in the sham group (*p* < 0.01). The correlations between distal brain regions, such as the cortex/hippocampus, cortex/thalamus, cortex/hypothalamus, and cortex/amygdala, were low. High correlations were found within different local brain regions, such as within the cortex, thalamus, and hypothalamus (Figure 5A). In contrast, 274 (59.3%) significant positive correlations were found in the CPSP group (*p* < 0.01). The cortex had a positive correlation with the hippocampus, thalamus (but not VB subarea), hypothalamus, amygdala, and PAG (Figure 6A).

The correlations in the mPFC-amygdala pathway, STT, and MT-ACC pathway were further analyzed in the sham (Figures 5B, C) and CPSP (Figures 6B, C) groups. The correlations observed in the correlation matrix were manifested as the clustering of nodes that belonged to the cortex (blue nodes), thalamus (red nodes), hypothalamus (brown nodes), amygdala (green node), and PAG (orange node). The subregions of the mPFC (i.e., IL and PrL) had a significant correlation with the hypothalamus and amygdala when comparisons were made between the sham and CPSP groups (Figure 6B). The subregions of the ACC-thalamus pathway were positively correlated with the PrL, Cg1, secondary motor cortex (M2), S1, MD, VL, VMLH, and VMH (Figure 6C).

Altogether, the brain subregions in the CPSP group had a higher percentage of correlations compared with the same regions in the sham group, regardless of whether they were in the STT, mPFC-amygdala pathway, or MT-ACC pathway. These results indicate that rats with CPSP might exhibit strong activation of the mPFC-amygdala pathway. The high interregional correlations of subregions of the STT and MT-ACC pathway probably reflect complex connections between the thalamus and ACC.

### Group differences in inter-regional correlations of regional cerebral blood flow of neural substrates

**Figure 7:**
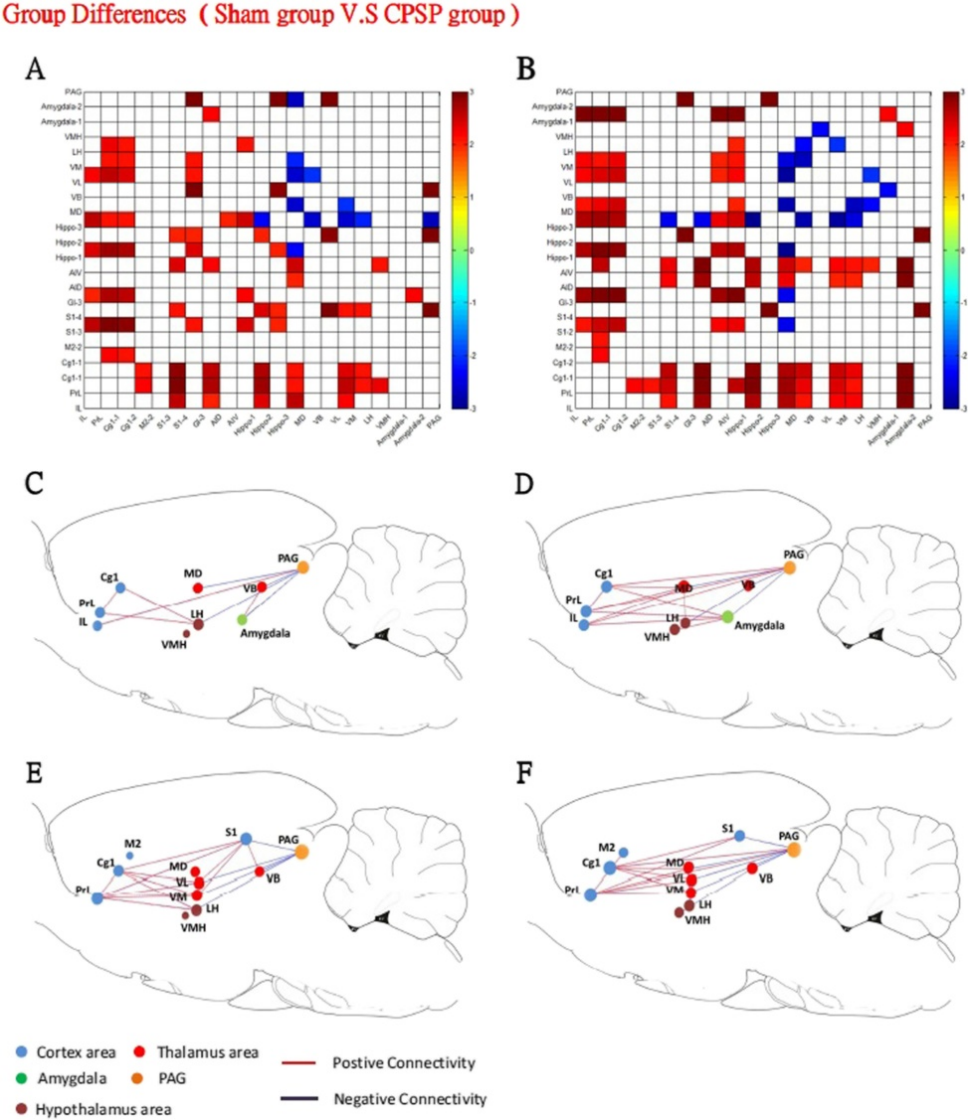
**Differences in inter-regional correlations of rCBF of neural substrates in the CPSP and sham groups. A and B**. Left and right brain connectivity. The matrix of Fisher’s Z-statistics represents differences in Pearson correlation coefficients (*r*) between the CPSP and sham groups. Positive Z values indicate a greater *r* in the CPSP group, and negative Z values indicate a smaller *r*. **C-F**. The inter-regional correlation of neural substrates is represented by a graph. The ROIs are represented by nodes, and significant correlations between different ROIs are represented by different line values. The red lines denote significant positive correlations, and blue lines denote significant negative correlations.

Brain activation patterns in the left and right hemispheres in the sham and CPSP groups are shown in Figures 7A and B, respectively. Figure 7A shows the matrix of Fisher’s Z-statistics, representing differences in Pearson correlation coefficients (*r*) between the CPSP and sham groups in the left hemisphere. A total of 114 significant correlations were identified (24.7%), of which 84 were positive (73.7%) and 30 were negative (26.3%) at the threshold of *p* < 0.05. Figure 7B shows the results in the right hemisphere. A total of 140 significant correlations were identified (30.3%), of which 104 were positive (74.3%) and 36 were negative (25.7%) at the threshold of *p* < 0.05.

The pattern in the left hemisphere appeared to be similar to the right hemisphere. High correlations were found among the cortex, hippocampus, thalamus, hypothalamus, and amygdala. Furthermore, within the subregions of the mPFC-amygdala pathway, the left hemisphere showed no correlations between the mPFC (such as the IL and PRL) and amygdala (Figure 7C). However, the IL and PRL of the mPFC were highly correlated with the amygdala in the right hemisphere (Figure 7D).

In the left hemisphere, the ACC (such as the PrL and Cg1) had low correlations with the VM, VL, and MD of the thalamus (Figure 7E). However, the right hemisphere showed complex connections among the subregions of the STT and MT-ACC pathway. For example, the PrL and Cg1 had positive correlations with the MD, VM, and VL of the thalamus (Figure 7F).

## Discussion

The effects of lateral thalamic lesions were evaluated in the present animal model. Lesions of the VB induced thermal and mechanical hyperalgesia and anxiety-like responses. The qualitative analysis revealed [^14^C]-IAP activation in the mPFC, ACC, thalamus, hypothalamus, amygdala, and PAG. The ROI analysis indicated that the IL, Cg1, M2, AID, AIV, Hippo-3, and PAG of the left hemisphere and IL, PrL, Cg1-1, M2, and amygdala of the right hemisphere were activated. Activation in the lesion site (i.e., the VB) was also significantly diminished in the right hemisphere (*p* < 0.05). Pain-related behavior in the plantar test was negatively correlated with the ROI data (*p* < 0.05). The CPSP group exhibited a stronger interregional correlation of rCBF among brain networks compared with the sham group (*p* < 0.05). The CPSP group exhibited significant correlations in the mPFC-amygdala pathway, STT, and MT-ACC pathway (*p* < 0.05). The comparison of the left and right hemispheres in the CPSP and sham groups showed that the neural subregions of the mPFC-amygdala pathway, STT, and MT-ACC pathway were significantly correlated in the right hemisphere compared with the left hemisphere in the CPSP group (*p* < 0.05). The present results indicate that rats with VB lesions exhibit symptoms of CPSP following hemorrhagic stroke. The behavioral tests revealed a lower nociceptive threshold in response to thermal and mechanical stimulation in the VB lesion group (*p* < 0.05). The present [^14^C]IAP analysis indicated that the mPFC, ACC, thalamus, hypothalamus, amygdala, and PAG were more activated than other brain structures in CPSP. The inter-regional analysis of correlations of rCBF showed that the STT, MT-ACC pathway, and amygdala-mPFC pathway were significantly activated. To our knowledge, no previous study has investigated brain activation and CPSP using the [^14^C]IAP approach. Our [^14^C]IAP uptake findings in CPSP, however, are not entirely consistent with previous [^14^C]IAP studies that used an animal model of acute pain. For example, colorectal distension elicited significant enhancements in rCBF in the insula cortex, somatosensory cortex, ACC, and amygdala, but the thalamus, parabrachial nucleus, PAG, hypothalamus, and pons showed decreases in rCBF [27]. Furthermore, a study that used a passive avoidance conditioned learning paradigm with noxious colorectal distension to induce visceral pain demonstrated that rats with acute pain conditioning exhibited brain activation in the anterior insula, somatosensory cortex, ACC, PrL, and amygdala [28]. Although the experimental paradigms used different animal models of pain, the ACC and amygdala were both shown to be common brain areas involved in acute and chronic pain.

The ACC is an important part of the medial pain pathway. Previous studies showed that brain areas in the STT and MT-ACC pathway, including the ACC, are implicated in CPSP [7,11,12]. With regard to the involvement of the STT and MT-ACC pathway in CPSP, the lateral thalamus was thought to disrupt the inhibition of signaling to the MT, resulting in mechanical allodynia and thermal hyperalgesia [6,7]. This impairment of inhibition has been shown to result from lesions of the STT, producing thalamic hyperexcitability at the neural level, thermal hyperalgesia, and mechanical allodynia [27]. Clinical studies have also shown that the STT and MT-ACC pathway, particularly the ACC, mediate pain responses in CPSP patients. For example, CPSP patients who were subjected to different thermonociceptive stimuli and underwent fMRI showed pain-specific signal changes in the anterior cingulate gyrus and associative parietal regions [24]. A recent clinical brain mapping study suggested that the ventral posterior nucleus and pulvinar border zone were involved in CPSP, but the VPL was not [15,17]. Therefore, basic and clinical studies of CPSP suggest that the MT-ACC pathway and STT play crucial roles in CPSP.

Our inter-regional correlation of rCBF analysis showed that subregions of the mPFC (e.g., IL, Cg1, Cg2, and PrL) were more connected to the amygdala in the CPSP group. The qualitative [^14^C] analysis and quantitative ROI analysis also indicated that mPFC nuclei, such as the IL and PrL, were also involved in CPSP. Therefore, the amygdala and mPFC may both mediate part of the symptoms of CPSP. According to previous data [19-23], the mPFC might be a high-level controller of cognition that elaborates or regulates emotional content from the amygdala, whereas the amygdala governs the emotional response to pain [29-31]. Additionally, the mPFC and amygdala might play different functional roles. For example, the administration of D-cycloserine, an *N*-methyl-D-aspartate receptor partial agonist, into the mPFC or amygdala reduced neuropathic pain in a spared nerve injury model [32]. The glutamatergic system may be involved in antinociception in the mPFC-amygdala pathway, and other neurotransmitters may mediate the nociceptive effect in the mPFC-amygdala pathway [33].

The present findings of the involvement of the PAG in CPSP appear to be consistent with previous studies with regard to the crucial role of the PAG in descending control [34-36]. For example, a recent study that used electrophysiological recordings suggested that noxious and innocuous cold information was transmitted through descending systems via the PAG to process acute or chronic pain [34]. A behavioral pharmacology study showed that microinjection of lidocaine, a sodium ion channel inhibitor, into the PAG impaired noxious information transfer from the medial or central amygdala, and reflected by decreases in the latency of the tail flick reflex [35]. Furthermore, a recent study demonstrated that oxytocin perfusion into the PAG decreased pain thresholds, indicating that the PAG may be involved in antinociceptive processing through oxytocin systems [36]. Therefore, the PAG might also play an important role in descending modulatory control in CPSP.

**Figure 8:**
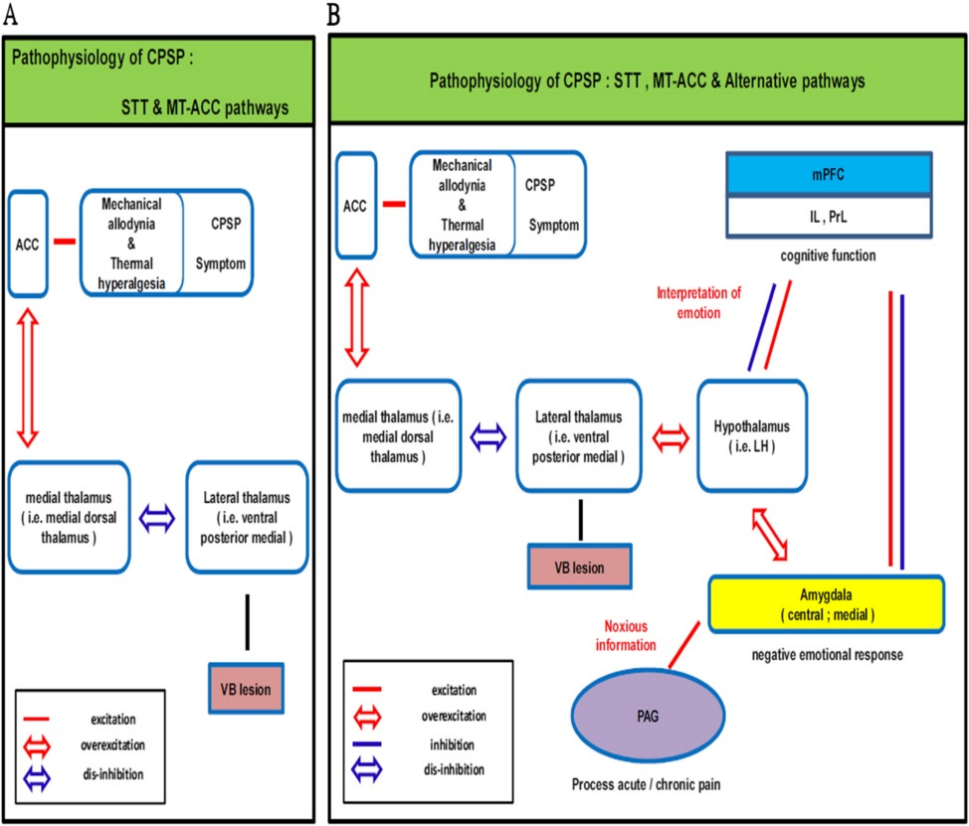
**Neural network hypothesis related to the present animal model of CPSP. A**. Pathophysiological mechanisms in the MT-ACC pathway and STT are triggered to disinhibit the projection from the lateral thalamus and insula to the medial thalamus, resulting in mechanical and thermal hyperalgesia. **B**. The mPFC plays an executive function role to interact with the amygdala, which responds to negative emotional information. The mPFC inhibits activation of the amygdala, whereas negative information in the amygdala is conveyed to the mPFC. The mPFC then interprets the emotional information properly. Nociceptive information from the amygdala is transmitted to the PAG to process acute and chronic pain to induce antinociception.

A neural correlation hypothesis based on our animal model of CPSP may be proposed that involves changes in various structures, pathways, and circuits (Figure 8). The neural correlation hypothesis of CPSP-induced posits that pain symptoms are associated with spontaneous pain rather an acute pain, which would be different from conventional pain theories. CPSP is likely mediated by multiple pathophysiological mechanisms that involve alterations in various neural substrates. When the VB is damaged in hemorrhagic stroke, pathophysiological mechanisms are triggered in the STT and MT-ACC pathway, in which projections from the lateral thalamus (including the VPM) and insula to the medial thalamus (e.g., the MD) are disinhibited, thus resulting in mechanical and thermal hyperalgesia (Figure 8A) [6-8]. Alternative neural pathways, including the mPFC, amygdala, and PAG, were activated during the pathophysiological changes (Figure 8B). The mPFC, including the PrL and IL, plays a role in executive function and interacts with central or medial nuclei of the amygdala that respond to negative emotion. The mPFC might inhibit activation of the amygdala, whereas negative information from the amygdala is conveyed to the mPFC and mPFC to interpret the emotional information properly. Concurrently, nociceptive information from the amygdala is transmitted to the PAG to process acute and chronic pain and induce antinociception.

The neural correlation hypothesis of CPSP may have implications for clinical interventions. For example, pharmacological and nonpharmacological approaches can be applied to activate the neural circuits of the mPFC and PAG and inhibit amygdala activity. Hemorrhagic insult triggers a series of pathophysiological changes in pain-related brain areas, including cortical and subcortical structures. Such CPSP-associated changes also trigger a series of brain disorders, such as emotional fear, anxiety, depression, and impairment of cognitive function. Elucidation of the alterations in brain activation patterns and circuit connectivity in CPSP could provide insights into the extensive pathophysiological mechanisms that underlie the multi-faceted manifestations of CPSP. Effective therapeutic interventions would rely on a better understanding of the complex relationship between pain and emotional disorders and identify the cellular and molecular targets that contribute to these processes.

An interesting issue is why the occurrence of CPSP alters the relationship between the plantar test and von Frey test results and functional brain imaging. According to the data presented in Table 2, the von Frey and plantar test results were in the same direction in the sham group. In the CPSP group, in contrast, the von Frey and plantar test results presented a significant negative correlation. The correlation data also showed that the von Frey test result showed a nonsignificant correlation with the ROI brain imaging data. Different sensitivity of nociceptive responses has been reported in CPSP patients [7]. In the present study, behavioral responses in the von Frey test after CPSP did not show a negative correlation. This issue of differential nociceptive sensitivity in CPSP should be investigated in further studies.

## Conclusions

The present data were consistent with the previous findings that the STT and thalamocingulate pathway play a crucial role in the pathophysiology of CPSP symptoms. The mPFC, amygdala, and PAG were involved in important connectivity related to pain processing in CPSP. The present data may have implications for novel CPSP treatments.

## Methods

### Experimental animals

Fourteen male Sprague–Dawley rats (300–400 g) were housed in an air-conditioned room (21-23°C; 50% humidity; 12 h/12 h light /dark cycle, lights on at 8:00 AM) with free access to food and water. All of the experiments were performed in accordance with the guidelines of the Academia Sinica Institutional Animal Care and Utilization Committee.

### Surgical procedures

The rats were initially anesthetized with 4% isoflurane (in 100% O_2_), and body temperature were maintained at 36.5-37.5°C with a homeothermic blanket system (Model 50–7079, Harvard Apparatus, Holliston, MA, USA). The rats were randomly and equally assigned to the CPSP (*n* = 7) and sham (*n* = 7) groups. The CPSP rats were injected with 0.125 U of type 4 collagenase in 0.5 μl saline in the VPM and VPL (coordinates: 3.0-3.5 mm posterior and 3.0-3.5 mm lateral to bregma, 5.5-5.8 mm depth) [26]. The sham animals were injected with 0.5 μl sterile saline in the same region. The animals were injected with antibiotic (6 mg/kg gentamycin, i.p.) to prevent infection, and they were weighed before and after the lesion procedure. Only animals whose body weights recovered after surgery were included in the experiment. At week 4, the rats were subjected to the same anesthesia procedure. To prepare for radiotracer infusion in awake, freely moving rats, the right external jugular vein was isolated and catheterized with polyethylene-50 tubing. A port at the terminal end of the tube was subcutaneously tunneled and fixed to the back skin. The animals were allowed to recover for 7 days before the last behavioral test at week 5 and radiotracer infusion. To prevent blockage of the tube, it was flushed every other day after surgery with 0.3 ml of 0.9% saline, followed by 0.1 ml of saline with 20 U/ml heparin.

### Behavioral testing

To measure mechanical and thermal nociceptive responses, von Frey and plantar tests were applied on the bilateral hind paws before the lesion procedure and 7, 14, 21, 28, and 35 days after the collagenase injection. Persistent pain behavior was evaluated in the open field test.

### von Frey test

In this study, mechanical hyperalgesia was assessed by measuring the withdrawal response to a mechanical stimulus using a von Frey esthesiometer. The animals were placed on an elevated mesh platform for 30 min before testing, which provided access to the plantar surface of the hind paw. A rigid tip attached to the meter was applied to the plantar surface from under the floor, and filaments were gradually applied with ascending, graded force to determine the minimal force that would elicit a withdrawal response. The threshold was defined as the average of three minimal forces measured in consecutive trials, each separated by 5 min.

### Plantar test

Thermal hyperalgesia was assessed by measuring the hind paw withdrawal latency in response to radiant heat using a plantar test apparatus (IITC 390G Plantar Test, IITC Life Science, Woodland Hills, CA, USA). Each rat was placed in a transparent Plexiglas box for 30 min before testing, and the mobile infrared source was delivered through the glass floor. The hind paw was directly stimulated by the infrared light source to assess withdrawal responses, giving withdrawal latencies of 0–20 s. The latency of the withdrawal response was the time that elapsed between pressing and releasing the button for infrared stimulation. Each rat was tested in three trials with the right and left hind paws, respectively. The intertrial interval was 5 min.

### Radiotracer injection and brain perfusion

On day 35 after the behavioral test, a syringe was filled with the radiotracer [^14^C]IAP (125 μCi/kg in 0.3-0.5 ml; American Radiolabelled Chemicals, St. Louis, MO, USA). The radiotracer-filled tubing was then connected to the animal’s cannula. The other end of the cannula was connected to a syringe filled with euthanasia agent (3 M potassium chloride). After resting for 5 min, anesthesia-free rats were infused with the radiotracer at a rate of 2.25 ml/min through the external jugular vein. The animals were euthanized with 4% isoflurane for gas anesthesia and 0.5 ml of 3 M potassium chloride (i.v.) approximately 10 s after the cessation of radiotracer infusion. The brains were rapidly removed 1 min after euthanasia, quickly frozen in dry ice/methylbutane (approximately −55°C), and embedded in Optimal Cutting Temperature compound (Sakura Fintek, Torrance, CA, USA).

### Autoradiography

Brain mapping was performed using an autoradiographic method [37,38]. Perfusion autoradiography was conducted for the whole-brain assessment of brain activation in awake, unrestrained animals. A 10-s time window for the radiotracer injection provided the specific timing for [^14^C] uptake in brain tissue. This time condition of the radiotracer injection is appropriate for evoked pain [39]. However, the spontaneous pain associated with CPSP is assumed to be constant and persistent pain. The 10-s time window for the radiotracer injection is also suitable for measuring spontaneous pain associated with CPSP. Therefore, the brain images showed the signal strength of [^14^C] radioactivity. The uptake of the [^14^C] radiotracer was determined by measuring the pixel intensity in selected ROIs. The signal of the pixel intensity is expressed as pixels per square millimeter. To normalize the images obtained from brain slices from different rats, the ROI pixel intensity was transformed to radioactivity count based on the method of Brandt et al. [40]. The signal of the radioactivity count is expressed as the beta count per square millimeter.

The amount of [^14^C] in brain tissue was calibrated using densitometric measurements. The first step was to establish the relationship between the pixel intensity and radioactivity count. Five different doses of [^14^C] (0.001, 0.01, 0.1, 1, and 10 μCi) were dropped onto 25 mm^2^ filter paper. The pixel intensities of each image of the filter papers were measured and correlated with the radioisotope count of each filter paper. Images were obtained by exposure to exposure cassettes with a phosphor screen (Amersham Biosciences, Piscataway, NJ, USA) at −20°C and digitized using 8-bit gray scale in Typhoon 9410 Variable Mode Imager (WS-S9410, GMI, Ramsey, MN, USA). The radioactivity count was obtained using a γ-counter (Beckman LS 6500 Liquid Scintillation Counter, Beckman Coulter, Brea, CA, USA) to evaluate the radioactivity count of the filter papers. Furthermore, the data were conducted a liner regression analysis for the pixel intensity and radioactivity count, and then Equation 1 (Y = 824.48 X −23020; see Figure 1) was obtained.

In the second step, brain slices were prepared, and the radioisotope counts of the brain tissues were estimated from Equation 1 by calculating the pixel intensity of the ROI image. Coronal slices (20 μm thick) were sectioned on a cryostat (Leica CM1850, Leica Biosystems Nussloch GmbH, Nussloch, BW**,** Germany) at −20°C, and the interval between each slice was 240 μm. The slices were dried on glass slides and placed alongside five standard filter papers with graded radioactivity. All of the slides were exposed to Amersham Biosciences exposure cassettes at −20°C. The images of the brain sections were then digitized. To quantify the [^14^C] signal from the ROIs of the different rat brain slices, the pixel intensity obtained from each brain slice was normalized based on the value of the five standard filter papers, and the radioactivity count was calculated according to Equation 1 to transform the pixel intensity. The ROI signals were transformed into an *X* value using this equation, and these *X* values indicated the quantitative radioactivity counts. To determine the best exposure time, the brain slices were measured from 1 to 5 days.

### Data analysis

#### Statistical parametric mapping

Statistical Parametric Mapping (SPM, version 8, Wellcome Centre for Neuroimaging, University College London, London, UK), a software package developed for the analysis of functional brain imaging data, was adapted for the analysis of rat brain autoradiography. The pixel count was replaced by the pixel intensity, quantified autoradiographically, and analyzed using SPM and the ROIs, respectively. A three-dimensional brain image was reconstructed using 62 serial coronal sections (starting at bregma +4.8 mm). Adjacent sections were aligned both manually and using StackReg, an automated pixel-based registration algorithm in ImageJ software (version 1.46; http://imagej.nih.gov/ij). All of the original three-dimensionally reconstructed brains were smoothed and normalized to the reference rat brain model and divided into sham and CPSP groups. Following the formation of these two groups, all of the brains were averaged to create the final brain template. To determine significant differences between the images in these two groups, the images were derived by subtracting the sham group from the CPSP group. Finally, we chose key brain slice images to show significant differences in pixel counts between the CPSP and sham groups.

#### Region of interest analysis

The ROI was functionally defined as a set of pixel intensities of a brain area that showed [^14^C] uptake in brain tissue. A total of 63 anatomical ROIs (31 on each side of the brain plus the cerebellum as a reference ROI) were first depicted in ImageJ over the template brain according to the rat brain atlas [41], and the following brain regions were measured: cortex (IL, PrL, Cg2, Cg1, M2, M1, S1, GI, AID, AIV), striatum, Hippo, thalamus (MD, VB, VL, and VM), hypothalamus (LH and VMH), amygdala, and PAG. To quantify and make comparisons between the ROIs from the different groups, ROI signals were normalized to obtain the ratio of radioactivity in each ROI. The ratio was obtained by the following Equation 2:

2 $$ Radioactivity\  ratio=\frac{\left(X-{X}_b\right)-\left({X}_{ref}-{X}_b\right)}{\left({X}_{ref}-{X}_b\right)} $$

In Equation 1, the mean radioactivity value *X* was transformed from Equation 2 from the ROI of the different rat brain slices. *X* equals the mean radioactivity signal of the ROI. *X*_*b*_ equals the mean signal of radioactivity of the selected background region. *X*_*ref*_ is the mean signal of radioactivity of a reference ROI (i.e., the cerebellum). Pearson correlations were performed for pain-related behavior (including the plantar test and von Frey test) and ROIs of selected brain areas, respectively.

### Inter-regional correlation of regional cerebral blood flow analysis

Two different statistical analyses were performed to determine the inter-regional correlations of rCBF. To clarify the relative contribution of the ratio of the ROIs in each group, a correlation coefficient analysis was first performed, and the data were displayed in an inter-regional correlation matrix. The next part of the analysis used Pearson correlation coefficients to examine the relationships between the CPSP and sham groups. An inter-regional correlation matrix was calculated across animals from each group using MatLab (version 2009b, MathWorks, Natick, MA, USA) and visualized as color maps. The correlation coefficients were transformed into Z scores using equation of Fisher transformation, Equation 3 [28]:

3 $$ Z=\frac{\frac{1}{2} \ln \frac{1+{r}_1}{1-{r}_1}-\frac{1}{2} \ln \frac{1+{r}_2}{1-{r}_2}}{\sqrt{\frac{1}{n_1-3}+\frac{1}{n_2-3}}} $$

*r*_1_ and *r*_2_ are the Pearson coefficients in the CPSP group and sham group, and *r*_1_ is greater than *r*_2_.

Graphical theoretical analysis was performed on networks defined by the above correlation matrices using Pajek software (version 3.06; http://Pajek.imfm.si/). In the graph, a brain area is represented by a node, and a significant correlation between the two nodes is linked by edges. The nodes were arranged so that neighboring nuclei were placed closer to each other, and more distal nuclei were placed further apart. The Z scores of the correlation coefficients were used to define the strength of the edges, and edges that were thicker indicated that the Z scores had major significant differences.

### Statistical significance inferences

A 2 × 5 two-way mixed ANOVA, with group and weeks as factors, was conducted to assess the duration of heat tolerance in the plantar test and mechanical force in the von Frey test in the sham and CPSP groups in the pretest and at weeks 1–5. When appropriate, Tukey’s HSD *post hoc* test was conducted. For the quantitative SPM and radioactivity ratio analysis, a 2 × 31 × 2 (group *vs*. brain area *vs*. hemisphere) three-way ANOVA was conducted. A 2 × 31 two-way ANOVA was also conducted, with group and brain area as factors. When appropriate, Tukey’s HSD *post hoc* test was conducted. Pearson correlation coefficients were calculated to assess the correlations among all of the selected brain areas in the sham and CPSP groups across the whole subject pool. However, the ROI data of selected brain areas were further analyzed using the Pearson correlation test after determining significant brain areas in the ANOVA (i.e., a correction method to replace the whole brain areas). Moreover, the matrix of Fisher’s Z-statistics was analyzed using Pearson correlation coefficients between the sham and CPSP groups in the left and right hemispheres. Values of *p* < 0.05 and 0.01 were considered statistically significant in the ANOVA and Pearson correlation tests, respectively.

## Abbreviations

ACC: Anterior cingulate cortex
AID: Dorsal agranular insular cortex
AIV: Ventral agranular insular cortex
ANOVA: Analysis of variance
Cg1: Cingulate cortex area 1
Cg2: Cingulate cortex area 2
CPSP: Central post-stroke pain
fMRI: Functional magnetic resonance imaging
GI: Granular insular cortex
Hippo: Hippocampus
HSD: Honestly significant difference
IAP: Iodoantipyrine
IL: Infralimbic cortex
LH: Lateral hypothalamic area
M1: Primary motor cortex
M2: Secondary motor cortex
MD: Mediodorsal thalamic nucleus
MT: Medial thalamus
mPFC: Medial prefrontal cortex
PAG: Periaqueductal gray
PET: Positron emission tomography
PrL: Prelimbic cortex
QST: Quantitative sensory testing
rCBF: Regional cerebral blood flow
ROI: Region of interest
SPECT: Single-photon emission computed tomography
SPM: Statistical parametric mapping
S1: Somatosensory cortex
STT: Spinothalamic tract
VB: Ventral basal nucleus
VL: Ventrolateral thalamic nucleus
VM: Ventromedial thalamic nucleus
VMH: Ventromedial hypothalamic nucleus
VMpo: Posterior portion of the ventral medial nucleus
VPL: Ventral posterolateral thalamic nucleus
VPM: Ventral posteromedial thalamic nucleus
